# A Comparative Study of the Sedative Effect of Oral Midazolam and Oral Promethazine Medication in Lumbar Puncture

**Published:** 2013

**Authors:** Hojjat DERAKHSHANFAR, Mona MODANLOOKORDI, Afshin AMINI, Ali SHAHRAMI

**Affiliations:** 1 Associate Professor of Pediatric Emergency Medicine, Pediatric Department, Mofid Children Hospital, Shahid Beheshti University of Medical Sciences, Tehran, Iran; 2 General Physician, Education Development Center, Mazandaran University of Medical Sciences, Mazandaran, Iran; 3 Associate Professor of Pediatric Emergency, Pediatric Department, Imam Hossein Hospital, Shahid Beheshti University of Medical Sciences, Tehran, Iran; 4 Assistant Professor of Pediatric Emergency, Pediatric Department, Imam Hossein Hospital, Shahid Beheshti University of Medical Sciences, Tehran, Iran

**Keywords:** LP, Anxiety, Premedication

## Abstract

**Objective:**

Lumbar puncture (LP) essentially is a painful and stressful procedure, however indicated for diagnosis and therapeutic purposes. One way to reduce the anxiety is to administer an oral premedication. The aim of this study is to compare clinical effects of oral midazolam and oral promethazine in LP.

**Materials & Methods:**

This prospective randomized controlled clinical trial study was performed on 80 children aged 2-7 years that were candidate for LP. They were divided into two randomized equal groups. First group received oral midazolam syrup 0.5 mg/kg and the other group received oral promethazine syrup 1mg/kg. Level of sedation, hemodynamic changes and any other complications were monitored every 5 minutes from 30 minutes before the start of the procedure.

**Results:**

Midazolam group and promethazine group were similar in age, gender and weight. Midazolam had significantly shorter onset of sedation and also shorter duration to maximal sedation. The two groups were similar with respect to sedative effect at all time. The only complication that was significantly more in midazolam group was nausea and vomiting.

**Conclusion:**

Midazolam syrup and promethazine syrup have same sedative effect in children. Both of these medications are easy to use in preschool children and none of them appeared to be superior to another.

## Introduction

Lumbar puncture (LP) is indicated for both diagnostic and therapeutic purposes. It is used to obtain a sample of cerebrospinal fluid (CSF). Analysis of CSF is useful in the diagnosis of infectious processes and neurologic diseases. Therapeutically, LP can be used for subarachnoid injection of chemotherapeutic agents, anesthetic drugs, and antibiotics (1).

LPs are essentially painful and in some children can cause severe anxiety and distress (2-4) that can continue for months after the procedure (5).

The three main modalities reported for decreasing the preoperative anxiety in children are behavioral preparation programs of various kinds, parental presence during induction of anesthesia (PPIA), and sedative premedication (6,7).

Due to some limitations on behavioral preparation programs (8) and some findings about parents’ presence during induction (8,9), it can be concluded that these are not suitable substitutes for sedative premedication. 

Anxiolysis and sedation with oral midazolam are common practice in both adult and pediatric anesthesia because of its onset time of 10-20 min, short duration of action (30 min), and no complications at low doses (less than 0.5 mg/kg) (10,11).

Promethazine as an anti histamine (H1) agent has some sedative and antiemetic properties (12). This medication has been reported as an effective and safe sedative drug with low complications (5).

As a routine in Iran, the drugs most commonly prescribed by physicians for premedication are promethazine and midazolam. In the present study we aimed to assay the benefits of these two drugs to help the children and their parents and to provide them more satisfactory condition.

## Materials & Methods

This is a prospective randomized controlled clinical trial conducted in Emergency Department of Mofid Hospital in Tehran. The study group consisted of 80 children older than 2 years old, presenting for LP. Study design was approved by Ethics Committee of Mofid Children Hospital. All patients’ parents gave their written informed consent before being included in the study.

Patients were randomly separated in two groups. Patients of the first group received oral midazolam syrup 0.5 mg/kg, 50 minutes before LP (9) and in the second group, patients received oral promethazine syrup 1 mg/kg, 90 minutes before the procedure (5). 

Patients who had a hypersensitivity reaction or known idiosyncrasy to the study medications or having a history of psychiatric disease, or aged less than 2 years were excluded. 

Gutstein et al.’s five-point sedation scale (1992) was used in both groups to evaluate the children’s sedation levels (5,13), 30, 25, 20, 15, 10 and 5 minutes before the LP.

This scale included the following items:

1. Barely arousable (asleep, needs shaking or shouting to arouse)

2. Asleep (eyes closed but arouses to a soft voice or light)

3. Sleepy (eyes open, less active, with drawn)

4. Awake

5. Agitated

The last evaluation was at the time of the LP. At the same time patient’s heart rate, respiratory rate, blood pressure, and oxygen saturation percentage were recorded. 

An expert nurse was responsible for recording of any side effects in patients in each group.

After data collection, statistical analysis was performed with SPSS version18 using independent t-test to compare the groups. A p-value of<0.05 interpreted as statistically significant.

## Results

The mean age in the midazolam group was 4.2+2.1 years (range 2.1-6.7) and it was 4.6+1.8 years (range 2.3-6.5) in promethazine group, (p<0.831). The mean weight was 17.2+4.7 kg (range 10.8-31.2 kg) in midazolam group and 17.0+5.2 kg (range 10.1-30.0 kg) in promethazine group (p<0.144). There were 25 girls and 15 boys in midazolam group and 23 girls and 18 boys in promethazine group. There was no statistically significant difference with respect to gender between the two groups (p<0.071). Two groups were similar with respect to age, gender and weight.

Onset of sedation was 25.5+12.2 (12-50) min in midazolam group and 60.55+15.5 (40-90) min in promethazine group (p<0.001). Peak sedative effect was observed at 34.3+11.2 min for midazolam and at 79.5+16.4 for promethazine (p<0.022). These differences were both statistically significant.

There was no significant difference in sedation score between two groups (Table 1). 

No significant occurrences of hemodynamic changes occurred in either group (Table 2).

The only side effect that was more with midazolam than with promethazine included nausea and vomiting (p<0.014). The other complications of the drugs were not significantly different between the groups (Table 3).

**Table 1:** Sedation Score, Midazolam Compared With Promethazine

Time	Midazolam group	Promethazine group	p-value
Basal	4.91	4.87	<0.471
30 min	4.68	4.76	<0.528
25 min	4.22	4.18	<0.079
20 min	3.68	3.76	<0.917
15 min	3.06	3.20	<0.345
10 min	2.28	2.33	<0.915
5 min	1.98	1.98	<0.147
Just before the procedure	1.94	1.96	<0.257

**Table 2:** Hemodynamic Changes, Midazolam Compared With Promethazine

	Heart rate			Respiratory rate			O_2_ saturation	
Time	Midazolam group	Promethazine group	p-value	Midazolam group	Promethazine group	p-value	M group	P group
basal	128.7	119.7	>0.123	26.8	25.3	>0.514	>95%	>95%
30 min	126.3	120.3	>0.345	26.2	25.1	>0.620	>95%	>95%
25 min	118.7	118.6	>0.384	25.8	24.6	>0.291	>95%	>95%
20 min	117.8	117.3	>0.08	25.2	23.9	>0.191	>95%	>95%
15 min	115.3	116.5	>0.626	24.9	24.1	>0.310	>95%	>95%
10 min	114.4	115.2	>0.563	24.3	23.4	>0.151	>95%	>95%
5 min	112.2	114.2	>0.831	23.8	22.6	>0.274	>95%	>95%
Just before the procedure	111.3	113.6	>0.186	23.1	21.8	>0.161	>95%	>95%

**Table 3:** Complications of The Drugs, Midazolam Compared With Promethazine

**group **	**Midazolam (%)**	**Promethazine (%)**	**p-value**
**complication**			
Nausea and vomiting	3 (7.5)	1 (2.5)	<0.012
Headache	4 (10)	4 (10)	1
Agitation	3 (7.5)	5 (12.5)	<0.114
Prolonged sedation	8 (20)	7 (17.5)	<0.095
No complication	22 (55)	23 (57.5)	<0.124

## Discussion

Lumbar puncture has a critical role in diagnosis and therapy of some disease, so in many cases this practice is inevitable (1). Considering this fact, in this study, we compared the sedative effects of oral midazolam and oral promethazine in some children presenting for LP to evaluate the sedative dimensions of the two drugs. 

Pharmacologic advantages of midazolam which we found included a rapid onset of action and a shorter duration to maximal sedation that can accelerate patients’ recovery.

This finding is similar to that of Mathai et al. (2011) that compared nasal midazolam and oral promethazine on 100 preschool children in the preoperative period (5). Also in another study by Almenrader et al. who compared oral midazolam and oral clonidine, they found that oral midazolam needs shorter time to achieve onset of sedation and also peak sedative effect (14). 

As in Mathai et al.’s study, we found no difference between the levels of sedation induced by oral midazolam and oral promethazine (5).

On the contrary, in some other studies, the results were different (15,16). Singh et al. concluded that oral midazolam is a better sedative drug compared to promethazine and oral triclofos (15). Same as them, Naziri et al. in a study on 56 children presenting for elective surgery, showed that for separation children from their parents, oral midazolam was preferred to oral promethazine. But their population included children under 5 years of age (16).

On the other hand, some other studies were in contrast with them. Parkinson et al. and Crean et al. showed that a combination of promethazine and chloral hydrate is better than midazolam for children’s sedation in PICU (17,18).

Similar to Mathai et al. and Naziri et al. findings, we did not find any significant differences in hemodynamic changes between our two groups (5,16). Also, in a recent study it was shown that midazolam doesn’t affect mean blood pressure and heart rate before and during surgery (19).

We did not detect any severe adverse events among children. Except nausea and vomiting, there was not any significant difference between complications which followed introduction of two drugs. Promethazine is one of the most frequent drugs used for treatment of nausea and vomiting in the world (20), and this is a natural expectation that children will have less nausea in recovery phase after sedation with promethazine.

The main advantage of our study in the comparison with other studies was that our population were not candidate for surgeries and they did not need induction of general anesthesia. Therefore, we could assess actual amount of sedative medications without the interference effect of anesthetic drugs.

However, there were several limitations to our study. Our sample size was small. In addition, we only studied children older than 2 years old, because promethazine is contraindicated in children younger than 2 years old. So, our findings cannot be applied to children younger than 2 years of age.

Although, both midazolam and promethazine have same sedative effects in children, each of them have an advantage over another. Shorter onset of sedation and short duration to peak sedation can be considered in the outpatient setting when we want to use midazolam as a sedative drug. But on the other hand, a better recovery with less nausea and vomiting is the advantage of antiemetic promethazine prescription. 

Both drugs are easy to use (oral administration) for preschool children and we conclude that either of them can be used for reduction of the anxiety before and during the LP. 

However, the writer recommends that multicentre studies should be done on a greater population with more varieties of sedative drugs.
